# Breast Mass in a Rubens Painting

**DOI:** 10.5041/RMMJ.10243

**Published:** 2016-04-19

**Authors:** Davide Lazzeri, Donatella Lippi, Manuel Francisco Castello, George M. Weisz

**Affiliations:** 1 Plastic Reconstructive and Aesthetic Surgery, Villa Salaria Clinic, Rome, Italy; 2 Center for Medical Humanities, Department of Experimental and Clinical Medicine, University of Florence, Italy; 3 School of Humanities, University of New England, Armidale, NSW, Australia; 4 School of Humanities, University of New South Wales, Sydney, Australia

**Keywords:** Baroque, breast mass, medico-artistic, Rubens

## Abstract

Deformity of the breast and axilla observed in famous paintings is a fascinating field for the medico-artists. The attempt of a retrospective diagnosis of breast tumors is highly challenging. This paper deals with a Rubens painting portraying the heroine Judith with a visible but previously unreported left breast mass. Though speculative, the present medico-artistic diagnosis is of a tumor likely to be of benign nature. It is of interest that the present case is the sixth breast disease discovered in Rubens’s works.

## INTRODUCTION

The increasing realism pursued by artists following the Renaissance revolution has made observers today expect art works to be truthful. Depiction of certain deformities was always present due to the artists’ styles in reproducing human figures. A medical diagnosis within the artistic field, such as those discussed in this paper, and in the referenced ones,^1^–^4^ can only be provided by direct visual evaluation, rarely assisted by technological tools. Such diagnosis remains presumptive, unless a written document presents a description and the intention of the painter.^4^

Peter Paul Rubens belongs to the Baroque movement. He fully embraced the philosophy of extreme realism in portraits representing biblical or mythological female bodies, including breasts. They represent particular interest in the detection of breast tumors.^1^,^3^ A portrait dated 1617 constitutes the subject of this paper, with particular focus on the model’s left breast.

## THE PAINTING

**Figure 1:**
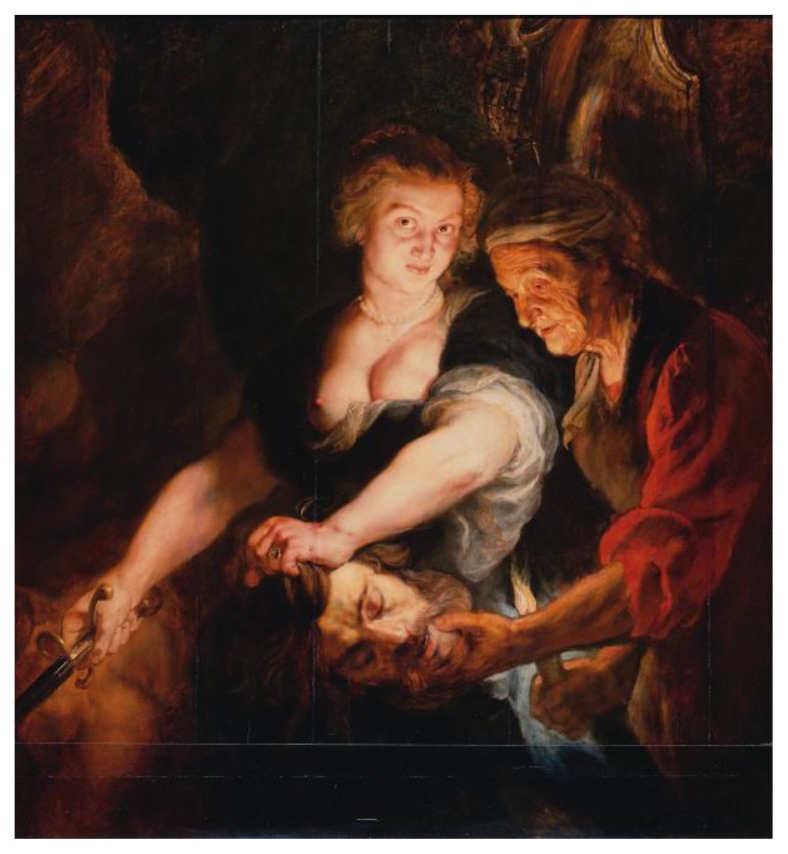
*Judith with the Head of Holofernes (Book of Judith 13:11)*, Peter Paul Rubens (1617), oil on panel, 120×111 cm (47.2×43.7 in). On display at the Herzog Anton Ulrich-Museum, Kunstmuseum des Landes Niedersachsen, Braunschweig, Germany. Peter Paul Rubens [Public domain], via Wikimedia Commons.

The story of *Judith and Holofernes* is given in the deutero-canonical “Book of Judith” and is a recurrent topic in Renaissance and Baroque paintings (Figure 1). Judith was a beautiful chaste widow, coming from Bethulia. The city was besieged by the Assyrian army guided by Holofernes, who had fallen in love with Judith. She entered the General’s tent, overcame him with drink, and decapitated Holofernes. Her facial expression is of hatred and disgust, and the image, although aesthetic, is quite violent.

**Figure 2:**
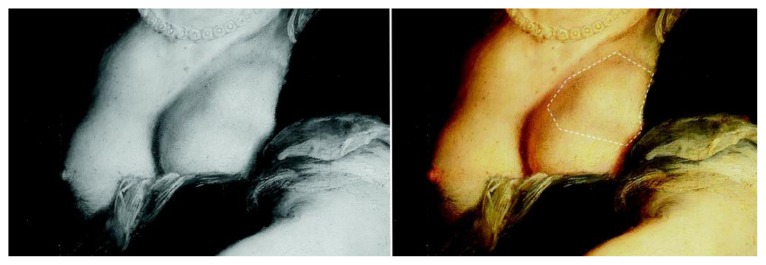
Close-up of Figure 1. Left: The mass is highlighted in the grey scale pattern. Right: the boundaries of the mass are identified by the dotted white line.

In this painting Rubens depicted Judith’s left breast with a lump involving the upper lateral quadrants, extending toward the subclavicular and axillary areas (Figure 2). No skin discoloration or ulceration is present, no redness or swelling is visible, the tumor is soft, multi-loculated, with non-retracted skin, and no skin surface alteration or hematoma signs are present. The nipple-areola complex is not visible.

## DISCUSSION

Because of Rubens’s high reputation, an inaccurate depiction of the model would be unlikely. Indeed, a breast mass would not have been intentionally drawn. In accordance with Rubens’s pursuit of realism, it is our opinion that he realistically painted the mass in the model’s breast. The attempt of a retrospective diagnosis of breast tumors in classic paintings is highly challenging. There are four aspects commonly raised in establishing a diagnosis.^1^–^3^,^5^

### Existent Imaging

We observe that the breast tumor is soft, lobulated, irregular at the margins, with no skin retraction, no skin surface discoloration, nor “peau d’orange” present. Visually it does not correspond with infection or traumatic hematoma, it is not uniform, it is with irregular margins, and it does not have the features of a malignancy.

### The Artist’s Intention

An intentional, rather than inadvertent inclusion of a deformed breast in a beautiful woman (likely the muse or the lover of the painter himself) makes no sense. Why would the painter not include a perfect model? Why wouldn’t he replace, if recognized, a “sick” image with a “healthy” representation?

### Differential Diagnosis

Uncommon breast conditions such as Mondor’s disease^3^ or mammary deformities with typical aspects of malignancy have been previously detected in Rubens’s paintings involving two of his Three Graces, Delilah, Eurydice, and Diana pursued by the satyr.^1^ Despite the visible difference with the benign tumor herein reported, these findings confirm the familiarity of Rubens in the depiction of his models’ breasts and his extreme realism. There is no known written diagnosis in any of the Rubens portrayed cases, including in this “*Judith with the Head of Holofernes*.”^1^,^2^ Indeed, we have no way to correlate the depicted image with clinical signs. Our diagnosis is of benign disease, with a differential diagnosis among giant cyst, fibrocystic disease, giant fibro-adenoma, adenosis, phyllodes tumor, hamartoma, lipoma, hemangioma, adenomyoepithelioma, neurofibroma, and pseudoangiomatous stromal hyperplasia.

### Recognition of the Model

**Figure 3:**
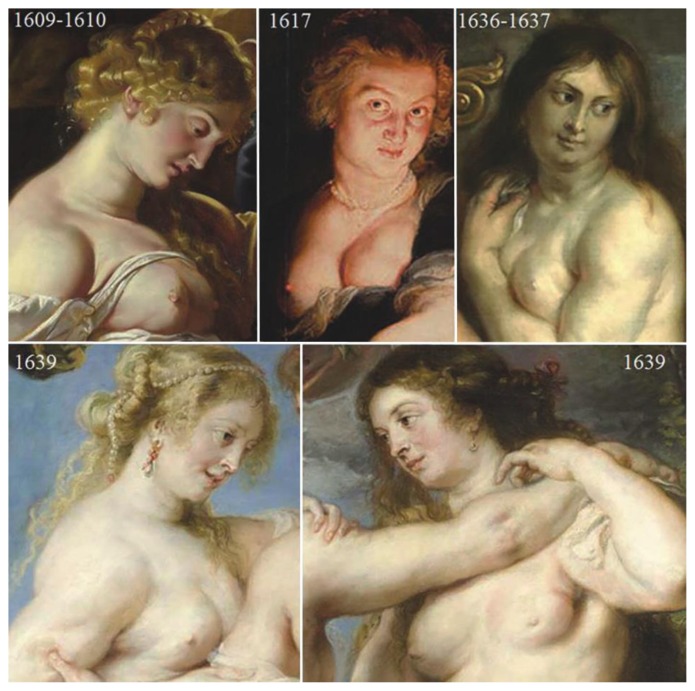
A collation of all models depicted by Rubens in which breast diseases have been recognized. **Top left**: Detail of *Samson and Delilah*, Peter Paul Rubens (1609–10), oil on wood, 205×185 cm (81×73 in). On display at the National Gallery, London, UK. Peter Paul Rubens [Public domain], via Wikimedia Commons. **Top center:** Detail of *Judith with the Head of Holofernes*, Peter Paul Rubens (1617), oil on panel, 120×111 cm (47.2×43.7 in). On display at the Herzog Anton Ulrich-Museum, Kunstmuseum des Landes Niedersachsen, Braunschweig, Germany. **Top right:** Detail of *Orpheus and Eurydice*, Peter Paul Rubens (1636–7), oil on canvas, 245×194 cm (96.4×76.3 in). On display at the Kunsthaus, Zürich, Switzerland. Peter Paul Rubens [Public domain], via Wikimedia Commons. **Bottom:** The left Grace **(left)** and the right Grace **(right)** of *The Three Graces*, Peter Paul Rubens (1639), oil on canvas, 221×181 cm (87×71 in). On display at the Prado Museum, Madrid, Spain. Peter Paul Rubens [Public domain], via Wikimedia Commons.

A collation of all the models depicted by Rubens in which breast diseases have been recognized (Figure 3) does not allow any clear identification of the sitter of the portrait here referenced.

Art historians give credit to the theory that the Fourment sisters were used as models.^3^,^6^ Hélène Fourment (the second wife of Rubens) served partly as the model for numerous portraits, including the left Grace. She was born in 1614, and her sister Susana in 1599, so we should exclude that Rubens used his second wife or his sister-in-law as models for the Judith.

**Figure 4:**
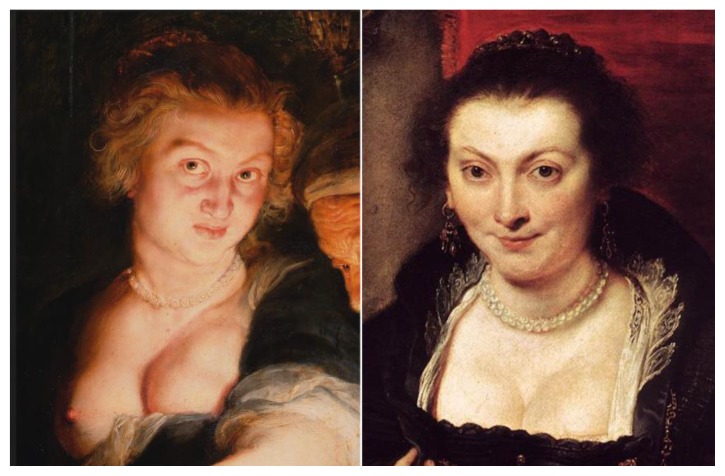
A Comparison between the *Judith with the Head of Holofernes* (left) and the *Portrait of Isabella Brant* (right) by Rubens (*c.*1625), oil on canvas. On display at the Galleria degli Uffizi, Florence, Italy. Portrait of Isabella Brant by Peter Paul Rubens [Public domain], via Wikimedia Commons.

Instead, the first wife of Rubens, Isabelle Brant, has facial features similar to the Judith (Figure 4). She died in 1629 of plague 12 years after the Judith depiction, an event that would support our theory about the benign nature of the mass. There is no portrait other than Figure 4 (right) in which the breast of Isabelle may be evaluated. Rubens or his contemporary art historians did not leave any written documents that could contribute in this debate.

## CONCLUSION

Mammary deformities observed in famous paintings represent a fascinating field of the medico-artistic diagnosis. The discussion is ongoing with lots of controversies. The present investigation deals with a painting in which the sitter shows a breast mass, visually assessed as being of a benign nature. This medico-artistic diagnosis is speculative, but likely correct. It is of interest that the present case is the sixth breast disease discovered in Rubens’s works.
